# Patterns, control and complications of diabetes from a hospital based registry established in a low income country

**DOI:** 10.1186/s12902-017-0179-1

**Published:** 2017-06-05

**Authors:** Jaweed Akhter, Asma Ahmed, Minaz Mawani, Laila Lakhani, Ayaz Kalsekar, Shehla Tabassum, Najmul Islam

**Affiliations:** 1 SETMA Diabetes Center, Beaumont, TX USA; 2 Aga Khan University, Karachi, Pakistan; 3 Section of Endocrinology, Department of Medicine, Aga Khan University, Karachi, Pakistan; 4 Department of Medicine, Aga Khan University, Karachi, Pakistan; 5 UT Houston Internal Medicine, Houston, USA; 6 Endocrine & Diabetes Section, Department of Medicine, The Aga Khan University Hospital, Stadium Road, P.O. Box 3500, Karachi, 74800 Pakistan

## Abstract

**Background:**

Diabetes registry enables practitioners to measure the characteristics and patterns of diabetes across their patient population. They also provide insight into practice patterns which can be very effective in improving care and preventing complications. The aim of this study was to assess the patterns, control levels and complications at the baseline of the patients attending clinic at the large tertiary care hospital in Karachi, Pakistan with the help of the registry. This can be used as a reference to monitor the control and also for a comparison between peer groups.

**Methods:**

This was a cross sectional study with the data obtained from diabetes registry collected with the help of pre-designed questionnaire. HbA1c was used as a central diabetes measure and other related factors and complications were assessed with it.

**Results:**

Only 16.6% of the participants had optimal HbA1c ≤ 7.0%. 52.9% of the patients were classified as having poor control defined by HbA1c of >8%. Three fourth of the study population were obese according to Asian specific BMI cutoffs and majority had type 2 diabetes with duration of diabetes ranging from less than one to about 35 years, mean(SD) duration being 7.6 years (7.1). Overall only 4% of the patients were on combine target of HbA1c, LDL and BP. Results of multivariable logistic regression showed that the odds of having optimal glycemic control increased by 3% with every one year increase in age. In addition, having longer duration of diabetes was associated with 56% lower odds of having good glycemic control. Moreover, having higher triglyceride levels was associated with 1% lower odds of having good glycemic control.

**Conclusion:**

This highlights the large burden of sub optimally controlled people with diabetes in Pakistani population, a low income country with huge diabetes prevalence and ineffective primary health care system creating enormous health and economic burden.

**Electronic supplementary material:**

The online version of this article (doi:10.1186/s12902-017-0179-1) contains supplementary material, which is available to authorized users.

## Background

The rapidly increasing burden of diabetes throughout the world is alarming. According to the recent update by the International Diabetes Federation (IDF) 415 million adults have diabetes and this number is projected to increase to 642 million by 2040 [1]. Among different regions of the world, South Asia (SA) is found to have very high prevalence of dysglycemia of around 43% [2]. Epidemiological data suggests that the prevalence of diabetes in SA has accelerated in the past two decades and is mostly associated with the large scale urbanization and industrialization [3, 4]. Increasing rates of overweight & obesity are among the important reasons for rise in prevalence. The highest age-standardized prevalence in the region is estimated to be in Mauritius (22.3%) followed by Maldives (9.2%) according to the 2015 estimate by IDF [1]. Obesity is the main culprit which is most prevalent in this region [5].

Pakistan has an estimated 6.9 million people affected with diabetes according to IDF and this number is expected to increase to 14.4 million by the year 2040 [1]. South Asians are more likely to develop type 2 diabetes mellitus (T2DM) at younger ages and at lower BMI than other races [2, 6]. People in the SA region have more visceral adiposity which increases their risk compared to Caucasians [7]. Visceral obesity which is associated with insulin resistance is metabolically unfavorable and contributes to lipotoxicity [8].

Despite the efforts to control morbidity and mortality associated with the disease, the rate of chronic complications, remain high [9]. According to previous studies, the reported prevalence of macro vascular diseases like cardiovascular disease (CAD), peripheral vascular disease (PVD) and cerebrovascular disease (CVD) is 27.2%. In addition to it, the prevalence of micro vascular manifestations of chronic hyperglycemia including retinopathy, nephropathy and diabetic foot ulcers is 53.5% [10]. Complications of diabetes are related to chronic hyperglycemia represented by high HbA1c levels. Therefore, HbA1c goals are established in various parts of the world to control the complications of diabetes mellitus. The American Diabetes Association, International Diabetes Federation, Canadian Diabetes Association and Diabetes Australia recognize an HbA1c level of 7% although most recognize that goals need to be set according to individual circumstances [11]. The American Association of Clinical Endocrinologist, Korean and Malaysian guidelines consider 6.5% or less [12, 13]. There are various studies which have used different cut-offs of HbA1c levels to report prevalence of uncontrolled diabetes mellitus (UDM) at various tertiary care centers in Pakistan but the quality of data is questionable and does not reflect the general population. Khowaja et al using a cut-off level of ≥7.4% reported a prevalence of 51.6% of UDM in 2010 [14] while a recent study done at community based specialized care center at district central Karachi in 2014 reported prevalence of UDM of 39% using HbA1c cut-off level of ≥8% [15].

The metabolic and genetic profile of our population subset puts it at increased risk for certain complications compared to others [16]. There is a need to collect more accurate and uniform data regarding control and complications of diabetes in Pakistani population. Hospital based diabetes registries have been recognized as an important tool to evaluate the clinical course, outcome and complications of diabetes and to identify the high risk groups [17]. We established a diabetes registry in an outpatient setting for the patients attending diabetes clinic at a large tertiary care hospital in the city of Karachi which has an ethnically diverse population exceeding 25 million people. Our center is catering to the people belonging to different socioeconomic groups from the city and surrounding areas.

The aim of setting up a registry is to document characteristic and pattern of diabetes mellitus in our patient population. This registry was also set up to help physicians to monitor trends in care processes, risk factors, indicators and complications over time. The aim of the current study is to assess the patterns, control levels and complications at the baseline of the patients attending clinic with the help of the registry and this information can be used later on to measure the progress.

## Methods

### Study design and settings

This was a cross-sectional study with data obtained from a diabetes registry based in a large tertiary care hospital located in Karachi, Pakistan. The Aga Khan University (AKU) is a private tertiary care teaching hospital catering to the needs of population of Karachi and surrounding areas. It is accredited by the Joint Commission International. A diabetes registry has been established at the endocrinology clinics, AKU since 2014. It collects real time information on a predesigned form on all patients with diabetes mellitus patients presenting to the clinics. The protocol of the study was reviewed and given an exemption by The Aga Khan University Ethics Review Committee (ERC). Due to the retrospective nature of the study based on the guidelines by ERC, the need to take informed consent was waived. In addition, it is usually conveyed to patients at the time of their visit that the information regarding their health could be used for research purpose. The information was not collected on any personal identifiers. The files were accessed at the time of clinic visit so no additional permission was required.

### Selection of participants

This study captured all patients diagnosed with diabetes mellitus and presenting to the study site between 22^nd^ September 2014 and 31^st^ July 2015 for the first time. The data was collected retrospectively.

### Data collection procedures

A physician trained in data collection reviewed all medical record charts at the diabetes & endocrinology clinics, and entered data in the diabetes registry. Random checks were done by the investigators of the project to ensure integrity and accuracy of data collection.

The questionnaire (Additional file 1) used in registry consisted of five different sections; (i) Demographics and initial assessment (ii) medications (iii) Comorbid conditions/complications (iv) Physical examination (v) Laboratory data (vi) Management plan. It collects information on age, gender, BMI, smoking history, comorbid conditions, and years since diagnosis, current medications, micro and macro vascular complications and lab data was entered. Any undocumented information was referred back to physician who saw the patient during visit.

At the clinics, weight (kg) and height (cm) are measured using mechanical scales. The limits for BMI were taken according to Asian specific cut off highlighted by World Health Organization. The categories suggested for Asians are: less than 18.5 kg/m2 (underweight); 18.5–23 kg/m2 (normal); 23–27.5 kg/m2 (overweight) and 27.5 kg/m2 or higher (obesity). Blood pressure (BP) is checked using mercury sphygmomanometer or digital scale while patient is in sitting position and arm at the level of heart. All measurements are done as a part of initial assessment by trained nurses. A repeat blood pressure is checked by the doctor when patient goes inside the clinic for the assessment. All instruments undergo standard maintenance procedures at regular intervals as part of institutional quality assurance.

### Outcomes

On target/optimum glycemic control was the primary outcome of this study and was defined as HbA1c either less than or equal to 7.0. On target BP was defined as blood pressure <140/90. LDL was said to be on target if it was <100, combine target was defined as on target LDL, BP and HbA1c.

### Statistical analyses

Statistical analysis was carried out using statistical package for social sciences (SPSS, version 19.0). Mean with SD were reported for all quantitative variables with normal distribution such as age and frequency with percentages were reported for all categorical variables such as gender, smoking status, type of diabetes, comorbid conditions, complications etc. Chi-square test was used to explore characteristics across groups with on target HbA1c, BP and LDL. Independent sample *T*-test was used to compare quantitative variables such as Age, HbA1c etc. across categories. Logistic regression analysis was used to explore factors associated with optimum glycemic control (HbA1c ≤ 7.0). Crude ORs along with 95% Confidence intervals (CI) were calculated. Variables with a *P*-value ≤ 0.25 or biologically plausible associations were selected for multivariate logistic regression analysis. Adjusted ORs along with their 95% CIs were calculated from multivariate logistic regression model. All potential confounders and interactions were evaluated. A *P*-value of <0.05 was considered as statistically significant for multivariable model.

## Results

During study period, a total of 876 patients presented to Diabetes clinic for an initial visit with mean age of 53.1 ± 11.9 years. There were approximately equal number of male and female patients and majority were 36 years and above. Three fourth of the study population were obese according to Asian specific BMI cutoffs with duration of diabetes ranging from less than one to about 35 years, mean(SD) duration being 7.6 years (7.1). Only 16.6% of the participants had optimal HbA1c ≤ 7.0%. 52.9% of the patients were classified as having poor control defined by HbA1c of >8%. About 42.2% of the participants had BP (either systolic HTN >140 or diastolic >90 or both). More than half of the participants (58.7%) whose Vitamin-D levels were evaluated had a deficiency (≤20). Nephropathy, depression and diabetic foot were presented in 13.8, 6.3 and 1.6% patients respectively. About 7.4% of men gave history of erectile dysfunction. Eye examination revealed background and proliferative diabetic retinopathy (DR) in 6.5 and 1.3% respectively. About 13.4% were reported to have peripheral neuropathy and 1.4% had peripheral vascular disease.

**Fig. 1:**
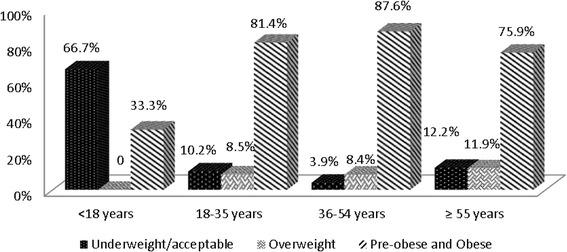
BMI levels by Age categories of initial patients presenting to diabetes

**Fig. 2:**
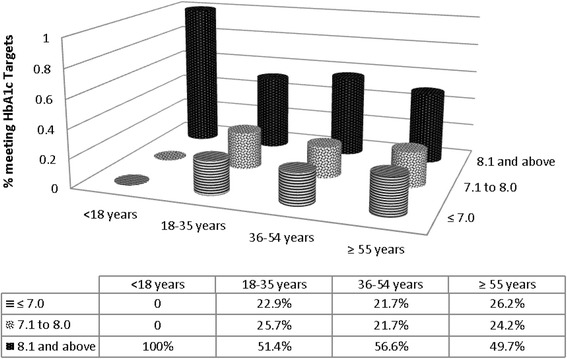
Percentage of participants having on target HbA1c on initial visit

Table 1 shows characteristics of the study participants stratified by age groups. An increasing trend was observed in percentage of obesity with age with a decline seen in participants with ages older than 55 years (*P* < 0.001). A higher percentage of patients in the age group 36-54 years had triglycerides more than or equal to 150 however in the later age this percentage is seen to be reduced (*P* = 0.003). Likewise, a higher number of participants in younger age group had higher LDL levels and a reducing trend was observed with increasing age (*P* = 0.01). Metabolic controls and BMI of study participants according to age categories are shown in Figs. 1 and 2.

**Table 1:** Demographic and clinical characteristics of initial patients presenting to diabetes clinics at AKU

Variables	Overall	<18 years old	18-35 years old	36-54 years old	≥55 years old	*P*-value
	*n* = 876	*n* = 3	*n* = 61	*n* = 396	*n* = 416	
Gender, n (%)						0.78
Male	454(51.8)	1(33.3)	34(55.7)	208(52.5)	211(50.7)	
Female	422(48.2)	2(66.7)	27(44.3)	188(47.5)	205(49.3)	
BMI, mean ± SD	29.6(5.6)	23.4(4.7)	29.9(6.7)	30.5(5.5)	28.7(5.2)	<0.001
Smoking status, n (%)						<0.001
Current smoker	85(9.8)	0(0)	6(9.8)	53(13.5)	26(6.3)	
Ex-smoker	81(9.3)	0(0)	3(4.9)	21(5.3)	57(13.8)	
Non-smoker	705(80.9)	3(100)	52(85.2)	319(81.2)	331(80.0)	
Duration of Diabetes, n (%)						<0.001
≤ 5 years	424(49.0)	2(66.7)	48(78.7)	240(60.9)	134(32.9)	
6-10	207(23.9)	1(33.3)	6(9.8)	91(23.1)	109(26.8)	
11-15	126(14.6)	0(0)	4(6.6)	42(10.7)	80(19.7)	
16-20	62(7.2)	0(0)	2(3.3)	16(4.1)	44(10.8)	
21-25	27(3.1)	0(0)	1(1.6)	4(1.0)	22(5.4)	
26-30	15(1.7)	0(0)	0(0)	1(0.3)	14(3.4)	
31-35	4(0.5)	0(0)	0(0)	0(0)	4(1.0)	
Comorbid conditions, n (%)						
Known HTN	486(55.5)	1(33.3)	18(29.5)	178(44.9)	289(69.6)	<0.001
Known Dyslipidemia	361(41.3)	0(0)	14(23.0)	158(39.9)	189(45.5)	0.003
Known CAD	116(13.3)	0(0)	0(0)	24(6.1)	92(22.2)	<0.001
HbA1c, mean ± SD	8.6(2.2)	11.3(2.4)	8.7(2.3)	8.7(2.1)	8.5(2.1)	0.17
LDL (mg/dl), n (%)						0.015
< 100	251(56.0)	0(0)	9(40.9)	102(50.2)	140(63.1)	
≥ 100(high)	197(44.0)	1(100)	13(59.1)	101(49.8)	82(36.9)	
HDL (mg/dl), n (%) Female						0.31
≥ 50(normal)	50(27.3)	0(0)	1(10.0)	18(23.7)	31(32.3)	
< 50(low)	133(72.7)	1(100)	9(90.0)	58(76.3)	65(67.7)	
Male						
< 40(low)	119(55.3)	0(0)	7(70.0)	63(58.9)	49(50.0)	0.28
≥ 40(normal)	96(44.7)	0(0)	3(30.0)	44(41.1)	49(50.0)	
Triglyceride (mg/dl), n (%)						0.003
< 150(acceptable)	218(50.3)	0(0)	10(47.6)	81(41.3)	127(59.1)	
≥ 150(high)	215(49.7)	1(100)	11(52.4)	115(58.7)	88(40.9)	
BP (mmHg), n (%)						<0.001
On Target(<140/90)	505(57.8)	3(100)	34(55.7)	256(64.6)	212(51.2)	
High(either >140 or 90 or both)	369(42.2)	0(0)	27(44.3)	140(35.4)	202(48.8)

A majority (59.5%) of the patients were on oral anti diabetic agents (OAD) alone, followed by 22.2% on combination therapy of OAD plus insulin, 10.8% on insulin alone and 7.6% of the participants were not on any pharmacotherapy for diabetes. About 7% of the participants were on treatment for neuropathy, 39.1% were on antiplatelet treatment, 40.1% on statins or other lipid lowering drugs and 52.6% on antihypertensive. A majority of patients receiving treatment for secondary prevention were > 55 years of age (Table 2). About 68.9% of patients were on metformin, 39.6% were on DPP4, 40.5% were on sulfonylureas, 3.8% were on glitazones, and 0.6% were on acarbose. About 255 patients were on one OAD, 292 were on 2, 160 were on 3 and 7 patients were on 4 OAD.

**Table 2:** Treatment data of initial patients presenting to diabetes clinics at AKU

Variables	Overall	<18 years old	18-35years old	36-54years old	≥55 years old	*P*-value
	*n* = 876	*n* = 3	*n* = 61	*n* = 396	*n* = 416	
Treatment, n (%)						<0.002
Insulin alone	94(10.8)	2(66.7)	5(8.3)	32(8.1)	55(13.3)	
OHA alone	520(59.5)	1(33.3)	33(55.0)	260(65.7)	226(54.5)	
Combine OHA and Insulin	194(22.2)	0(0)	15(25.0)	73(18.4)	106(25.5)	
Neither OHA nor Insulin	66(7.6)	0(0)	7(11.7)	31(7.8)	28(6.7)	
Lipid lowering agents (statins, fibrates, ezitimibe), n (%)						
Yes	351(40.1)	0(0)	7(11.5)	141(35.6)	203(48.8)	<0.001
No	525(59.9)	3(100)	54(88.5)	255(64.4)	213(51.2)	
Antihypertensive Agents, n (%)						<0.001
Yes	461(52.6)	1(33.3)	15(24.6)	175(44.2)	270(64.9)	
No	415(47.4)	2(66.7)	46(75.4)	221(55.8)	146(35.1)	
Antiplatelet, n (%)						<0.001
Yes	342(39.1)	0(0)	8(13.3)	108(27.3)	226(54.3)	
No	532(60.9)	3(100)	52(86.7)	287(72.7)	190(45.7)	
Pregablin/ gabapentin, n (%)						0.19
Yes	61(7.0)	0(0)	3(5.0)	21(5.3)	37(8.9)	
No	812(93.0)	3(100)	57(95.0)	374(94.7)	378(91.1)	

As compared to men, a higher percentage of women were obese (77.7% vs. 85.6%, *p* = 0.01) with longer diabetes duration (48.0% vs. 54.2%, *P* = 0.06), known history of hypertension (50.0% vs. 61.5%, *p* = 0.001), depression (3.5% vs. 9.3%, *p* < 0.001), elevated total cholesterol (18.6% vs. 26.9%, *p* = 0.04), elevated BP at clinic visit (38.3% vs. 46.4%) and low HDL levels (55.3% vs. 72.7%, *p* < 0.001). However, a higher percentage of men had elevated triglyceride levels i.e. ≥ 150(56.8% vs. 41.2%, *p* = 0.001), coronary artery disease (17.4 vs. 8.8, *p* < 0.001) and nephropathy (17.4 vs. 10.0, *p* = 0.001). Vitamin D deficiency was observed in about 66.7% of men as compared to 53.7% women (*p* = 0.04). There was no statistically significant difference in HbA1c alone or combine target of HbA1c, LDL and BP based on gender.

About 56% of the patients had on target LDL levels on initial visit. Those having on target LDL levels were significantly older, with longer duration of diabetes and had lower HbA1c. Most of the participants with on target LDL levels were on lipid lowering (61.8%), antihypertensive (65.3%) and antiplatelet (51.8%) drugs. About 58.5% of participants had on target BP. Patients with on target BP were younger, more likely to be males and with shorter duration of diabetes. A higher percentage of patients with elevated BP had peripheral neuropathy as well.

Overall only 4% of the patients were on combine target of HbA1c, LDL and BP. Those on target were significantly older with mean age 57.7 ± 13.8 vs. 52.8 ± 11.8, had acceptable total cholesterol (96.8% vs. 76.0%), LDL (100% vs. 52.3%) and triglyceride (75.8% vs. 48.3%) levels as compared to those not on target. About 71.4% of the patients who were on target were on lipid lowering agents, 82.9% were on OHA alone followed by 11.4% on no anti diabetics, 2.9% on insulin alone and 2.9% on combine OHA plus insulin. Gender, BMI, duration of diabetes, comorbid conditions and HDL levels were not significantly different across the categories.

About 23.9% of the patients had optimal glycemic control (HbA1c ≤ 7.0) on initial visit. Patients with optimal control were significantly older, with shorter duration of diabetes and lower percentage of known coronary artery disease as compared to patients with suboptimal glycemic control. A higher number of participants with suboptimal HbA1c also had elevated SGPT and triglycerides. A higher percentage of patients with optimal glycemic control were on OHA alone (71.7% vs. 54.7%) or not on any hypoglycemic agents (15.9% vs. 5.0%) and a lower percentage of these patients were on insulin alone (4.8% vs. 13.2%) and a combination of insulin and OHA (7.6 vs. 27.1) as compared to those with suboptimal control (Table 3).

**Table 3:** Factors associated with Glycemic control in patients presenting for an initial visit at AKU (*n* = 606)

Characteristics	Optimal Glycemic control HbA1c ≤7.0 145(23.9)	Suboptimal glycemic control HbA1c 7.1 and above 461(76.1%)	*P*-values
Age	55.9(12.8)	52.8(11.4)	0.007
Gender			0.50
Male	74(51.0)	250(54.2)	
Female	71(49.0)	211(45.8)	
BMI			0.61
Underweight/acceptable(<18.5 -22.9)	14(10.1)	33(7.5)	
Overweight(23-24.9)	14(10.1)	46(10.5)	
Pre-obese and Obese(25 and above)	110(79.7)	360(82.0)	
Smoking status			0.33
Current smoker	15(10.3)	44(9.6)	
Ex-smoker	9(6.2)	47(10.3)	
Non-smoker	121(83.4)	367(80.1)	
Duration of Diabetes, mean(SD)			0.01
≤ 5 years	85(59.9)	199(43.5)	
6-10 years	32(22.5)	107(23.4)	
11-15 years	16(11.3)	76(16.6)	
16-20 years	4(2.8)	44(9.6)	
21-25 years	3(2.1)	18(3.9)	
26-30 years	2(1.4)	9(2.0)	
31-35 years	0(0)	4(0.9)	
Comorbid Conditions			
Known HTN	84(57.9)	269(58.4)	0.92
Known Dyslipidemia	61(42.1)	206(44.7)	0.58
Known CAD	12(8.3)	76(16.5)	0.01
Complications			
Nephropathy	20(13.8)	86(18.7)	0.17
Depression	14(9.7)	30(6.5)	0.41
Diabetic foot	2(1.4)	10(2.2)	0.28
Amputation	1(0.7)	4(0.9)	0.37
Creatinine			0.69
< 1.5	114(93.4)	371(94.4)	
≥ 1.5	8(6.6)	22(5.6)	
SGPT			0.03
Normal(≤45 male, ≤35 female)	51(81.0)	143(67.5)	
High(>45 male, >35 female)	12(19.0)	69(32.5)	
Total Cholesterol			0.37
≤ 200 (normal)	73(82.0)	207(77.5)	
> 200 (high)	16(18.0)	60(22.5)	
HDL			0.11
Low(<50 for females, <40 for males)	48(54.5)	166(64.1)	
Normal(≥50 for females, ≥40 for males)	40(45.5)	93(35.9)	
LDL			0.17
< 100	62(63.9)	165(56.1)	
≥ 100(high)	35(36.1)	129(43.9)	
Triglyceride			0.09
< 150(acceptable)	54(59.3)	140(48.8)	
≥ 150(high)	37(40.7)	147(51.2)	
Vit B-12			0.35
< 150(deficient)	0(0)	3(6.4)	
150-201	2(10.0)	8(17.0)	
> 201(acceptable)	18(90.0)	36(76.6)	
Vit D			0.03
≤ 20.9(deficient)	11(40.7)	44(68.8)	
21-29.9(insufficient)	7(25.9)	6(9.4)	
30-150(sufficient)	9(33.3)	14(21.9)	
Urine Microalbumin			0.009
< 19(normal)	38(76.0)	69(54.8)	
≥ 19(high)	12(24.0)	57(45.2)	
Blood pressure			0.74
On Target(<140/90)	83(57.2)	271(58.8)	
High(either >140 or 90 or both)	62(42.8)	190(41.2)	
Fundoscopy			
Background DR	1(1.1)	24(7.8)	0.02
Proliferative DR	0(0)	4(1.3)	0.28
Foot Exam			
Peripheral neuropathy	13(10.4)	53(13.2)	0.41
Peripheral vascular disease	0(0)	10(2.5)	0.07
Treatment			<0.001
Insulin alone	7(4.8)	61(13.2)	
OHA alone	104(71.7)	252(54.7)	
Combine OHA and Insulin	11(7.6)	125(27.1)	
Neither OHA nor Insulin	23(15.9)	23(5.0)	
Lipid lowering agents(Statins, Fibrates, Ezitimibe)			0.40
Yes	61(42.1)	212(46.0)	
No	84(57.9)	249(54.0)	
Anti-hypertensive			0.71
Yes	83(57.2)	256(55.5)	
No	62(42.8)	205(44.5)	
Anti-platelets			0.15
Yes	52(35.9)	196(42.5)	
No	93(64.1)	265(57.5)	
Pre-gabalin			0.79
Yes	10(6.9)	35(7.6)	
No	134(93.1)	426(92.4)	

Factors associated with optimal glycemic control were examined using logistic regression models. On univariate level, Age (OR = 1.02; 95% CI = 1.00-1.03), higher duration of diabetes(OR = 0.51; 95% CI = 0.35-0.75), coronary artery disease (OR = 0.45; 95% CI = 0.24-0.86), higher SGPT levels(OR = 0.48; 95% CI = 0.24-0.97), higher triglyceride levels (OR = 0.65; 95% CI = 0.40-1.0), use of hypoglycemic agents(Insulin alone: OR = 0.11;95% CI = 0.04-0.30, OHA alone: OR = 0.41; 95% CI = 0.22-0.76, Combine insulin and OHA: OR = 0.08; 95% CI = 0.03-0.20) and presence of complications(OR = 0.60; 95% CI = 0.39-0.90) were found to be significantly associated with optimal glycemic control. In the multivariable model Age, duration of diabetes and triglyceride levels were associated with good glycemic control. After adjusting for other covariates, with every one year increase in age, the odds of having optimal glycemic control increased by 3%. Having duration of diabetes more than 5 years was associated with 56% lower chances of having good glycemic control. Higher triglycerides were associated with 1% lower odds of having good glycemic control. There was no confounding and interaction in the model (Table 4).

**Table 4:** Multivariable logistic regression analysis showing factors associated with good glycemic control among patients with diabetes

Variables	Adjusted Odds Ratio(95% confidence intervals)	*P*-value
Age	1.03(1.00-1.05)	<0.01
Diabetes Duration		
≤ 5	1	<0.002
> 5	0.44(0.26-0.74)	
Triglyceride		<0.03
< 150 (acceptable)	1	
≥ 150(high)	0.99(0.99-1.00)	

## Discussion

This study describes the baseline HbA1c in relation to certain clinical parameters of the patients presenting to the specialized diabetic clinic at tertiary care hospital in the large cosmopolitan city of Pakistan. The mean HbA1c was 8.6 ± 2.2% with duration of 7.6 ± 7.1 years and only 17% of the patients achieved glycemic goal of <7%,optimal target defined by ADA. This highlights the large burden of sub optimally controlled people with diabetes in our population causing enormous health care and economic burden. The mean HbA1c achieved is nearly comparable to the data reported previously from low and middle income countries of Asia and Middle East.

Poor glycemic control was associated with younger age, longer duration of diabetes and high triglyceride levels.

Overall, 58.7% of diabetics had insufficient vitamin D levels. The association of poor glycemic control with vitamin D deficiency has been controversial [18–20]. Based on our registry, no association was found between glycemic control and Vitamin D levels.

In terms of treatment of T2 DM, majority of the individuals were on oral anti diabetic agents and 16% were not on any form of treatment. On further analysis, 55% of diabetics on oral anti diabetic agents alone and 13.2% on once basal daily dose with OAD had suboptimal glycemic control. This can be attributed to the phenomenon of “clinical inertia” due to delivery of care by non-specialist and certain myths and misconceptions of the population regarding insulin treatment. The soaring and skyrocketing prices for insulin which has tripled between 2002 and 2013 could be another reason for the delay in treatment.

Macro vascular disease in the form of coronary heart disease, a primary cause of death in diabetes was found to be present in 13% of the individuals. Both micro and macro vascular diseases were more in patients with poor glycemic control.

This study also highlights the increasing prevalence of obesity in Pakistani population since two third of the patients presenting to diabetes clinic were obese based on south Asian targets of BMI. The majority of these obese individuals were in the middle age group (36-55 years of age) which increases the tendency to develop diabetes, HTN and lipid abnormalities in the most productive years of one’s life posing economic burden both at individual and societal level. The high BMI in majority of the individuals could also be the reason of not finding the association between poor glycemic control and BMI.

All the patients with poor glycemic control also had uncontrolled BP and lipid profile displaying the overall treatment inadequacy & paucity of control of other measures. Interestingly patients achieving optimal glycemic control did not meet the targets for lipid and blood pressure recommended by ADA. This was further shown to be mainly because of the underuse of medications for the control of lipids and BP since 62% of patients with dyslipidemia were not using any anti lipid medications. Likewise 40% of the hypertensive patients were not taking any anti-hypertensive treatment. This finding is of significant concern since these are the conventional modifiable risk factors which if not controlled can give rise to early onset of coronary heart disease and the risk is more in South Asians compared to Caucasians. The reasons could be multifactorial. It could be because of financial constraints since patient has to pay out of their pocket for these medications. The other reason could be the lack of adherence of the physicians to the treatment guidelines.

This study was limited by the fact that the data was acquired from the review of the charts completed by doctors at different levels. There are some factors which were not probably documented accurately like history of smoking which was noted down as positive in a minority of patients. Some of the complication data was based on the history and physical finding which could include some subjectivity.

Our registry has several strengths. First the data represents baseline characteristics of patients with diabetes from a large and diverse population belonging to different ethnic groups and socioeconomic levels. This is one of the few diabetes registries in Pakistan that would monitor the metabolic disease on regular basis.

With increasing prevalence of diabetes, the care provided at the basic primary level needs to be improved since majority of the individuals cannot bear cost of complications. Government and different non-governmental organizations should invest in polyclinics in different areas to provide cost effective care to diabetics. Indicators of quality care (HbA1c & other complications) should be assessed regularly for better diabetes management. Moreover patient registries should be used nationwide to improve the quality of the care provided to diabetic individuals.

## Conclusion

This registry confirms that large proportion of diabetic population do not achieve optimal level of glycemic control. In addition to the glycemic targets, the other parameters were also found to be not at target which can increase the risk of diabetic complications.

## Additional file

Additional file 1: Questionnaire used in the study. (PDF 1000 kb)

## Abbreviations

AKU: Aga Khan University
BMI: Body Mass Index
BP: Blood Pressures
CAD: Coronary Artery Disease
CVD: Cerebrovascular disease
DR: Diabetic Retinopathy
HbA1c: Hemoglobin A1c
HDL: High Density Lipoprotein
HTN: Hypertension
LDL: Low Density Lipoprotein
OAD: Oral anti diabetic agents
PVD: Peripheral Vascular Disease
SA: South Asia
SD: Standard Deviation
T2DM: Type 2 Diabetes Mellitus
UDM: Uncontrolled Diabetes Mellitus

## References

[1] www.diabetesatlas.org. DF Diabetes Atlas 2015(7th Edition).

[2] Shen J, Kondal D, Rubinstein A, Irazola V, Gutierrez L, Miranda JJ, Bernabe-Ortiz A, Lazo-Porras M, Levitt N, Steyn K (2016). A multiethnic study of pre-diabetes and diabetes in LMIC. Glob Heart.

[3] Mohan V, Seedat YK, Pradeepa R (2013). The rising burden of diabetes and hypertension in southeast asian and african regions: need for effective strategies for prevention and control in primary health care settings. Int J Hypertens.

[4] Cheema A, Adeloye D, Sidhu S, Sridhar D, Chan KY. Urbanization and prevalence of type 2 diabetes in Southern Asia: a systematic analysis. J Glob Health. 2014;4(1):010404. 10.7189/jogh.04.010404 PMC4073245 24976963

[5] Angkurawaranon C, Jiraporncharoen W, Chenthanakij B, Doyle P, Nitsch D. Urban environments and obesity in southeast Asia: a systematic review, meta-analysis and meta-regression. PLoS One. 2014;9(11):e113547. 10.1371/journal.pone.0113547 PMC4245122 25426942

[6] Ma RC, Chan JC. Type 2 diabetes in East Asians: similarities and differences with populations in Europe and the United States. Ann N Y Acad Sci. 2013;281:64–91. 10.1111/nyas.12098 PMC3708105 23551121

[7] Lear SA, Humphries KH, Kohli S, Chockalingam A, Frohlich JJ, Birmingham CL (2007). Visceral adipose tissue accumulation differs according to ethnic background: results of the Multicultural Community Health Assessment Trial (M-CHAT). Am J Clin Nutr.

[8] Chandalia M, Lin P, Seenivasan T, Livingston EH, Snell PG, Grundy SM, Abate N (2007). Insulin resistance and body fat distribution in South Asian men compared to Caucasian men. PLoS One.

[9] Basit A, Hydrie MZ, Hakeem R, Ahmedani MY, Masood Q (2004). Frequency of chronic complications of type II diabetes. J Coll Physicians Surg Pak.

[10] Litwak L, Goh SY, Hussein Z, Malek R, Prusty V, Khamseh ME. Prevalence of diabetes complications in people with type 2 diabetes mellitus and its association with baseline characteristics in the multinational A1chieve study. Diabetol Metab Syndr. 2013;5(1):57. 10.1186/1758-5996-5-57 PMC3854020 24228724

[11] Association AD (2016). Standards of medical care in diabetes—2016. Diab Care.

[12] Ko SH, Kim SR, Kim DJ, Oh SJ, Lee HJ, Shim KH, Woo MH, Kim JY, Kim NH, Kim JT (2011). clinical practice guidelines for type 2 diabetes in Korea. Diabetes Metab J.

[13] Garber AJ, Abrahamson MJ, Barzilay JI, Blonde L, Bloomgarden ZT, Bush MA, Dagogo-Jack S, DeFronzo RA, Einhorn D, Fonseca VA et al. Consensus statement by the American Association of Clinical Endocrinologists and American College of Endocrinology on the Comprehensive type 2 Diabetes Management Algorithm - 2016 executive summary. Endocr Pract. 2016;22(1):84–113. 10.4158/EP151126.CS 26731084

[14] Khowaja K, Waheed H. Self-glucose monitoring and glycaemic control at a tertiary care university hospital, Karachi, Pakistan. J Pak Med Assoc. 2010;60(12):1035–38. 21381559

[15] Siddiqui FJ, Avan BI, Mahmud S, Nanan DJ, Jabbar A, Assam PN. Uncontrolled diabetes mellitus: prevalence and risk factors among people with type 2 diabetes mellitus in an Urban District of Karachi, Pakistan. Diabetes Res Clin Pract. 2015;107(1):148–56. 10.1016/j.diabres.2014.09.025 25451895

[16] Ramachandran A, Ma RC, Snehalatha C. Diabetes in Asia. Lancet. 2009;375(9712):408–18. 10.1016/S0140-6736(09)60937-5 19875164

[17] Pollard C, Bailey KA, Petitte T, Baus A, Swim M, Hendryx M (2009). Electronic patient registries improve diabetes care and clinical outcomes in rural community health centers. J Rural Health.

[18] von Hurst PR, Stonehouse W, Coad J. Vitamin D supplementation reduces insulin resistance in South Asian women living in New Zealand who are insulin resistant and vitamin D deficient - a randomised, placebo-controlled trial. Br J Nutr. 2010;103(4):549–55. 10.1017/S0007114509992017 19781131

[19] Krul-Poel YH, Westra S, ten Boekel E, ter Wee MM, van Schoor NM, van Wijland H, Stam F, Lips PT, Simsek S. Effect of vitamin D supplementation on glycemic control in patients with type 2 diabetes (SUNNY Trial): a randomized placebo-controlled trial. Diab Care. 2015;38(8):1420–26. 10.2337/dc15-0323 25972575

[20] Kositsawat J, Freeman VL, Gerber BS, Geraci S (2010). Association of A1C levels with vitamin D status in U.S. adults: data from the National Health and Nutrition Examination Survey. Diab Care.

