# Supplementary material for: Patterns, control and complications of diabetes from a hospital based registry established in a low income country
Source: BMC Endocr Disord. 2017 Jun 5;17:30. doi: 10.1186/s12902-017-0179-1 (PMC5460467; doi:10.1186/s12902-017-0179-1)
Supplement: Additional file 1: — Questionnaire used in the study. (PDF 1000 kb) [file 12902_2017_179_MOESM1_ESM.pdf]

The Aga Khan University Hospital, Karachi
Department of Medicine

Patient Identification

Diabetes Progress Sheet

Date	Time	Dr. Name	wt	Ht	Pain Score	Sign
BP mmHg	Pulse /min		Resp /min		temp	

BMI: ________ Hip: ________ Waist: ________ Allergy: ________

Age: __________ Sex: M F Smoker: Y N Ex Initial / Follow up

Diabetes since _______yrs HTN ________ yrs Dyslipidemia ________ yrs. CAD _______ yrs

**Diabetic Medications**			**Other Medications**		
**Class**	**Type**	**Dose**	**Class**	**Type**	**Dose**
Insulin			Statins		
			ACE inhibitors		
Sulfonylurea			ARBs		
Metformin			Anti platelet		
Glitazones			Ezitimibe		
Acarbose			Beta Blockers		
others			Ca Channel blockers		
			Diuretics		

Other Complications / Comorbids:

Nephropathy ☐ Y ☐ N Diabetic Foot ☐ Y ☐ N HTN ☐ Y ☐ N

Dyslipidemia ☐ Y ☐ N S/P Amputation ☐ Y ☐ N CAD ☐ Y ☐ N

Depression ☐ Y ☐ N Erectile Dysfunction ☐ Y ☐ N

Examination:

Dental hygiene: ______

Fundoscopy ☐ Y ☐ N Background R ☐ Y ☐ N Proliferative retinopathy ☐ Y ☐ N

Foot exam ☐ Y ☐ N Peripheral neuropathy ☐ Y ☐ N Peripheral Vasc. Disesae ☐ Y ☐ N

Labs:

GLUF ______ GLUR _____ HbA1c _____ Creat ____ Na ____ K ____ SGPT ____

T. Cholesterol _____ LDL-Cholesterol ______ HDL Cholesterol _______ Triglycerides ________

Vit. B12 _____ Vit. D _____ TSH _______ Urine microalbumin ____

Subjective:

Objective:

Assessment:

Plan:

General Diabetes Education	
Diet & Exercise Education	
Foot care	
HBGM	
Insulin Techniques	
Insulin Pen Device	
Hypoglycemia Mx	

Sign & Mnemonic: ______________________
(Resident / Fellow)

Sign & Mnemonic: ______________________
(Consultant)
